# Aerial and ground locomotion of winged drones powered by a single actuator

**DOI:** 10.1038/s44182-026-00108-w

**Published:** 2026-07-29

**Authors:** Won Dong Shin, Hoang-Vu Phan, Simon L. Jeger, Tristan Bonato, Auke J. Ijspeert, Dario Floreano

**Affiliations:** 1 Bio-inspired Robotics and Design Laboratory, Pohang Science and Technology University, Pohang, Republic of Korea; 2 Laboratory of Intelligent Systems, École Polytechnique Fédérale de Lausanne, Lausanne, Switzerland; 3 Avian-Insect Robotics in Motion Laboratory, University of Nevada, Reno, NV USA; 4 Biorobotics Laboratory, École Polytechnique Fédérale de Lausanne, Lausanne, Switzerland

**Keywords:** Engineering, Physics

## Abstract

Multimodal drones combining aerial and terrestrial mobility offer adaptability and extended operational range across diverse environments. However, most existing multimodal drones rely on multiple actuators that add mass and complexity while offering limited terrestrial locomotion capabilities. Here, we introduce a multimodal winged drone driven by only a single actuator, capable of ground locomotion, flight, and ground-to-air transition by either rolling or jumping. The actuator is based on a novel transmission system that enables control of its rotational direction to switch between different locomotion modes. In one direction, the actuator drives a propeller that generates forward thrust for flight and (passive) wheeled locomotion on the ground, while in the other direction it activates a spring-leg mechanism that enables jumping by storing and releasing elastic energy. We show that the winged drone can perform fast wheeled locomotion on flat surfaces, consecutive jumps across diverse terrains, as well as take-off from a runway or jumping from a spot. Experimental characterization shows that runway take-off offers greater energy efficiency, while jumping take-off is more space-efficient and less dependent on ground conditions. The proposed actuation method enables simple and effective versatility for locomotion in diverse environments, thus extending the operational range of winged drones.

## Introduction

Flying animals, including birds and locusts, can navigate diverse environments by combining aerial and terrestrial locomotion. For long-distance travel, they rely on flight to reach their destinations quickly, while terrestrial locomotion through legs enables efficient navigation in confined areas with lower energy expenditure^1–9^. To transition from ground to air, these animals take off by jumping from a spot to generate the initial speed required for flight, regardless of the surrounding conditions^10–12^. Integrating aerial and ground locomotion with a jumping take-off strategy may provide flying robots with the adaptability to switch modes across diverse terrains. For instance, jumping take-off could free fixed-wing platforms from runway or launcher dependence and help overcome ground obstacles, as seen in birds and locusts^13–15^.

Nevertheless, unlike rotary^16–20^ or flapping wings^21–28^, jumping take-off is more challenging for fixed-wing vehicles^29–37^, as their wings are not able to actively generate initial aerodynamic forces for flight, and jumping requires extra appendages. The aerodynamic forces produced by wings depend on jumping speed and angle; insufficient speed or an improper angle can result in unsuccessful take-off^38^. Furthermore, adding appendages for multimodal locomotion increases mass and complexity of the drones^39^. The added mass may reduce flight efficiency or even prevent flight altogether. Due to these challenges, only a few multimodal winged drones can achieve a jump-based transition from ground to air, including jumping gliders^29–33^, jumping flappers^21,26,27^, and fixed-wing drones with bird-inspired jumping legs^35^. Most jumping gliders using a spring-loaded mechanism powered by a single actuator can jump off the ground and glide with their wings but cannot sustain powered flight^29–32^. However, adding extra flight components leads to a noticeable decline in the performance of jumping gliders^29,40^. Some large-scale flapping-wing drones are also able to take off by jumping, but are limited to a single jump^21,26^. In addition, their ground locomotion remains largely unexplored. Our recently described fixed-wing drone, RAVEN, with bird-inspired multifunctional legs demonstrated the abilities of jumping take-off, walking and hopping on the ground, and powered flight^35^. However, the ground versatility of RAVEN comes at the cost of complex leg designs with multiple independent actuators and controllers, considerably increasing the overall weight of the drone. A common problem in aerial-terrestrial robots is the increased mass resulting from the added complexity of multimodal design and the use of separate actuators for jumping and propulsion. In addition, although RAVEN demonstrated that jumping take-off is a fast and energy-efficient strategy when a runway is unavailable, the drone still spends a considerable amount of input power to initiate flight with the jump. When initiating flight from an extended flat surface, rolling or running to gain sufficient lift for take-off could be a more energetically efficient strategy – one that is also adopted by some large bird species^41^. However, no systematic comparison of jumping and runway take-off methods has been conducted.

**Fig. 1:**
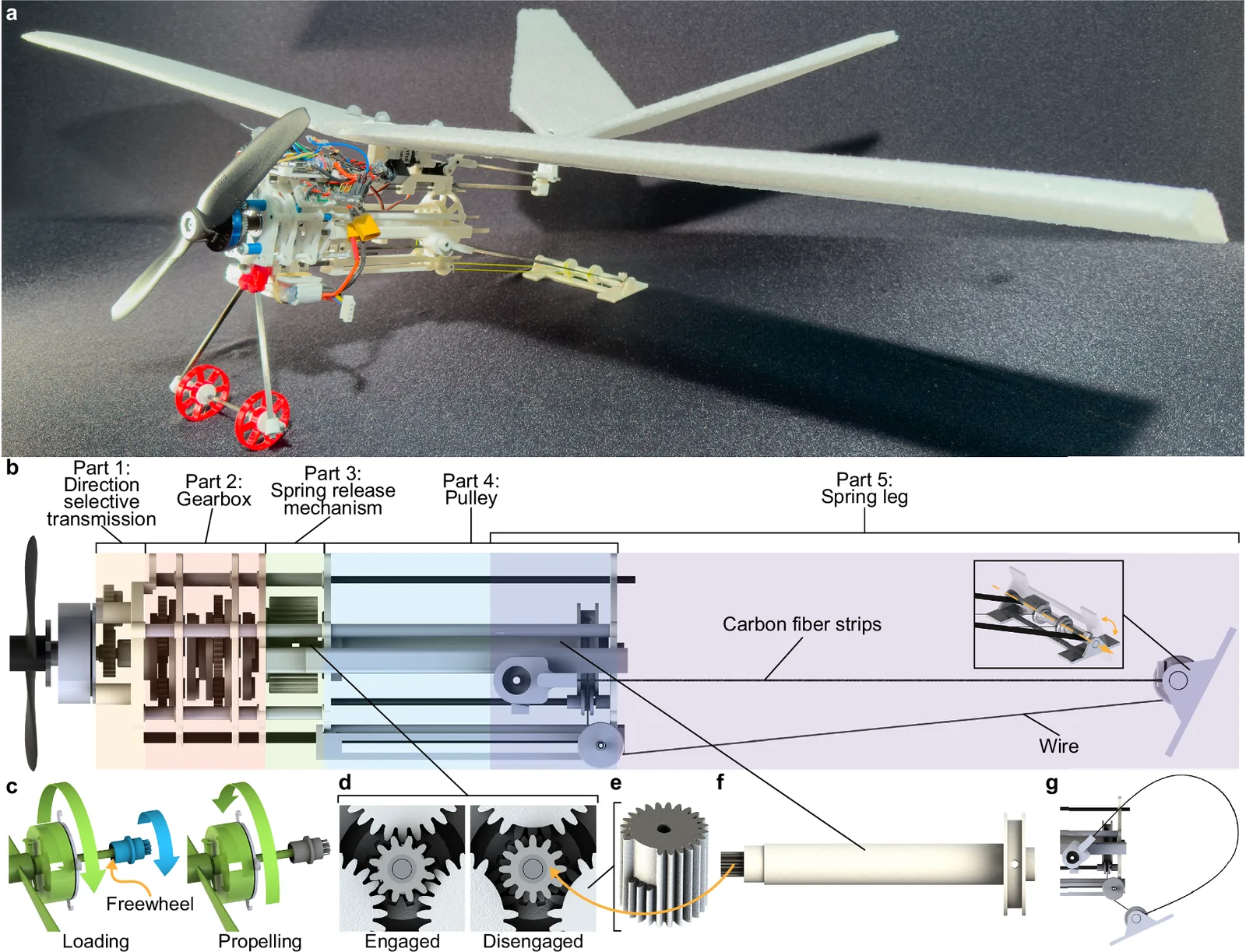
The multimodal drone is designed with a transmission mechanism that controls the rotational direction of the actuator to switch between modes. **a** Isometric view of the proposed platform. **b** Overview of the transmission system consisting of five parts. **c** Working principle of the direction-selective mechanism. With a freewheel, the motor transmits power to the gearbox (Part 1 in **b**) only when rotating clockwise (from the front view). **d** Spring release mechanism. The mechanism automatically releases the spring leg when the missing-tooth portions face the center. **e** Gear design with partially missing teeth. The bottom half of this gear is always engaged with the gearbox, while the top half repeatedly engages and disengages with the gear on the pulley due to the missing teeth. **f** Pulley design. The pulley is engaged with the spring release mechanism via the gear at one end. On the other end, a fishing wire passes through a hole and is tied to the foot (Part 5 in **b**). **g** Compressed configuration of the spring leg. When the pulley rotates, the fishing wire winds around it, and the carbon fiber legs bend to store energy as the distance between the pulley and the foot decreases.

Here, we introduce ATROSA (Aerial and Terrestrial RObot with Single Actuator), a multimodal drone driven by a single actuator that can locomote in aerial and terrestrial environments (Fig. 1a). Equipped with lightweight legs and wheels, ATROSA can travel on the ground by either jumping consecutively with legs or rolling with wheels. It can also transition into powered flight either by gaining sufficient speed with wheels or by jumping in the air. These capabilities are enabled by a transmission mechanism that switches locomotion modes based on the rotation direction of the actuator to either activate the legs for jumping mode or to generate propeller thrust for rolling and flight. For a jumping take-off, the actuator rotates clockwise to flex the legs that store energy and subsequently release it for jumping; upon detection of the powerful jump with an onboard Inertial Measurement Unit (IMU), the transmission rapidly reverses rotation direction and engages the propeller to power flight. Experimental results show that ATROSA can jump to take off and transition into flight from a constrained location. ATROSA is still capable of traditional runway take-off by accelerating on a clear surface with the propeller. A comparison between jumping and runway take-off methods with kinematic data and energy consumption data shows that runway take-off is more energy-efficient but requires a long and clear runway. The two take-off strategies thus complement each other, allowing ATROSA to adopt the method that is most effective depending on the environment. ATROSA also performed outdoor operation by seamlessly combining rolling, jumping, and ground-to-air transition to traverse ground obstacles, as well as performing flight and landing. Our work shows that a single-actuator-driven system can still be a versatile platform supporting multiple locomotion modes across different environments.

## Results

### Single-actuator transmission system

The drone consists of a custom-built fixed-winged platform with passive wheels, lightweight spring legs, and the transmission mechanism (Fig. 1). This transmission mechanism can switch locomotion modes by directing actuator power either to the spring legs or to the propeller and thus enable multiple locomotion modes.

For jumping, the transmission system converts actuator power into elastic energy through a gearbox that amplifies torque, bending the spring legs by pulling a fishing wire attached to their tips. The wire automatically releases once a certain length has been pulled. Five distinct parts (Fig. 1b) make up the transmission system and are connected in series to achieve this jumping procedure. The first part (Fig. 1b) is the direction-selective transmission, including a freewheel to selectively transfer power from the actuator to either the jumping legs or the propeller. When the actuator rotates clockwise from the front view (Fig. 1c), it engages the jumping mechanism. However, when the actuator rotates counterclockwise, the freewheel disengages from the jumping mechanism and only the propeller is actuated to generate thrust. The second part is the gearbox, which amplifies the torque output of the actuator to compress the spring during the energy-loading process. We stacked two planetary gearboxes^35^ to achieve a compact design and a high gear reduction ratio of 1:366. The third, fourth, and fifth parts are the automatic spring release mechanism (Fig. 1d, e), the pulley (Fig. 1f), and the spring legs, respectively. The pulley is connected to the tips of the spring legs using a fishing wire. As the pulley rotates, it pulls the wire, causing the spring legs to bend and store elastic energy in preparation for the jump (Fig. 1g). The stored energy is automatically released after a specific number of rotations of the pulley by the automatic spring release mechanism. The release mechanism consists of a set of three identical, specially designed gears (Fig. 1d, e). The bottom halves of these gears are fully toothed and mesh with the gearbox, allowing continuous rotation during the jumping process. The top halves are partially toothless and mesh with the pinion gear on the pulley. When the pinion gear aligns with the toothless sections, the pulley disengages and spins freely, releasing the compressed leg and thus the stored energy to power the jump. The spring legs are made of lightweight carbon fiber strips, as commonly used in other robotic jumping platforms^26,30,42^. A foot is attached to the end of the leg via a rotational joint, allowing the leg to adjust its angle relative to the ground during jumps (Fig. 1b). We affixed a silicone pad to the bottom of the foot to increase friction with the ground during jumping. However, in the rolling mode, the foot is flipped upward to allow the robot to move on small wheels (Fig. 1b).

ATROSA has a total mass of 279.49 g (Table 1), where the aerodynamic surfaces represent the largest mass contribution (89.89 g, 32.2%). The next largest contribution comes from the onboard electronic components (84.95 g, 30.4%), followed by the motor and controller (29.16 g, 10.4%). When grouped by functionality, the components that enable jumping account for a total mass of 75.77 g (27.1%), which increases up to 104.93 g (37.5%) when the motor and controller shared by the two functionalities are taken into account. Components that enable flight account for a total mass of 126.1 g (45.1%), which increases up to 155.26 g (55.5%) when the motor and controller shared by the two functionalities are taken into account. Overall, the system mass is dominated by electronic components. The single-actuator strategy reduces mass by 10.4% at the present scale because the controller and motor are shared between jumping and flight locomotion. This benefit is expected to become more pronounced as the robot is further scaled up in mass, because actuator redundancy would otherwise impose a larger penalty, as actuators and controllers may constitute a larger portion of the overall system, as shown in RAVEN (235.9 g of motors and controllers out of a total mass of 623.2 g, or 37.9%)^35^.

**Table 1:** Mass breakdown of ATROSA

Part	Subpart	Mass (g)	Quantity	Subtotal (g)	Total (g)
Body stucture	Chassis (Swiss Composite CFK-Vierkantrohr 4 × 4 mm)	1.83	1	1.83	73.53
	Transmission	44.65	1	44.65	
	Electric components holding board	4.32	1	4.32	
	Wheels	7.05	1	7.05	
	Small pulleys	4.94	1	4.94	
	Bottom carbon	1.28	1	1.28	
	Side carbon	2.07	1	2.07	
	Side structure	6.69	1	6.69	
	Freewheel (HF0306-KF-L564)	0.7	1	0.7	
Electric components on body	Battery (BETAFPV 3S 300 mAh)	25.52	1	25.52	84.95
	Motor (T-Motor AT2303 KV1500 Short Shaft) + connector	19.80	1	19.80	
	Microcontroller (STM32 B-G431B-ESC1)	9.36	1	9.36	
	IMU (Seeed Studio XIAO nRF52840)	2.40	1	2.40	
	Battery holder	2.02	1	2.02	
	RC Receiver (FrSky X4R)	3.79	1	3.79	
	Current sensor (Polulu ACS724)	1.85	1	1.85	
	SD card reader (SparkFun microSD Transflash Breakout) + SD card	2.72	1	2.72	
	5 V Voltage converter (VR10S05)	3.79	1	3.79	
	Wires, pins, and connetors	13.70	1	13.70	
Aerodynamic surfaces	Wings	62.58	1	62.58	89.89
	Tail	11.09	1	11.09	
	Servo motor (MASTER S706 MG)	5.50	2	11.00	
	Propeller (GWS 8040)	5.22	1	5.22	
Legs	Pulley	10.75	1	10.75	31.12
	Leg	13.33	1	13.33	
	Foot	6.79	1	6.79	
	Foot spring	0.25	1	0.25	
Total robot mass (g)		279.49			

### Multiple locomotion modes with a single actuator

**Fig. 2:**
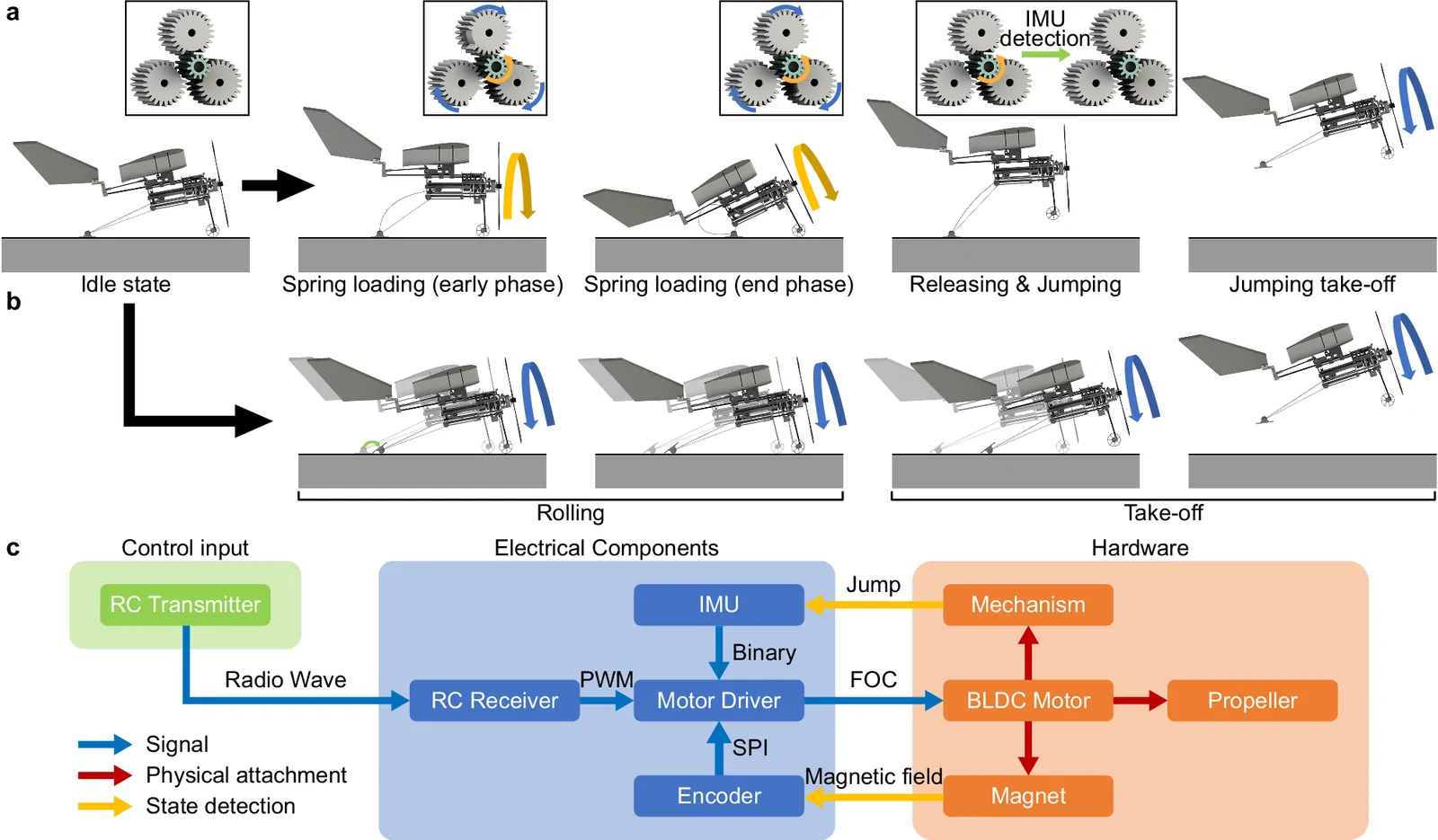
Take-off procedures and system architecture. **a** Jumping take-off procedure. To prepare for a jump, the motor rotates in the direction that stores elastic energy in the spring leg. Upon jumping, an embedded IMU detects the jump based on a change in forward acceleration, and the platform reverses the motor’s rotation direction to generate thrust. **b** Runway take-off procedure. The motor rotates only in the direction required to generate thrust. The platform can move on the ground. Once it accelerates and reaches the take-off speed, the drone automatically lifts off and transitions into flight. **c** System architecture. The system receives user input via radio waves from an RC transmitter. Based on the received signal, the motor driver actuates the BLDC motor, which is controlled in a closed-loop manner. The IMU detects forward acceleration, and if the acceleration exceeds a predefined threshold, the IMU sends a signal to the motor driver to switch the motor’s rotational direction.

The direction-dependent functionality of the transmission system enables ATROSA to switch between multiple locomotion modes by adjusting the actuator’s rotational direction and speed (Fig. 2a, b). To prepare for a jump, the actuator rotates clockwise (from the front view) to wind the fishing wire, bending the spring leg through the gear system (Fig. 2a). Once the spring release mechanism is triggered after a certain amount of actuator rotation, the spring leg automatically extends to push off the platform for a jump. This jumping motion can be used for ground obstacle clearance and repeated for consecutive jumps during ground traversal. Another locomotion mode available with the jump is the jumping take-off. With an embedded inertial measurement unit (IMU), ATROSA detects the jump and immediately reverses the actuator rotation to generate thrust and maintain flight after take-off (Fig. 2a). Counterclockwise rotation of the actuator generates forward thrust and enables two additional locomotion modes. This thrust allows ATROSA to roll on the ground, and when the rolling speed exceeds the take-off speed, ATROSA begins to lift off and perform a conventional runway take-off (Fig. 2b).

These various locomotion modes are controlled using a single-channel radio wave from a radio control (RC) transmitter (Fig. 2c). The radio wave is received by an onboard RC receiver embedded in ATROSA. Based on the radio wave input, the RC receiver sends a pulse-width modulation (PWM) signal to the motor driver, allowing the brushless direct current (BLDC) motor to determine which mode to activate and control its rotational direction and speed. A magnet attached to the motor works in conjunction with a magnetic encoder to track the motor’s rotation. This data is transmitted to the motor driver via a Serial Peripheral Interface (SPI) and used for feedback-based motor control. During a jumping take-off, the IMU detects a jump by sensing a change in the robot’s acceleration and sends a binary signal to the motor driver, which then reverses the motor’s rotational direction to generate flight propulsion.

### Simulated jumping take-off

**Fig. 3:**
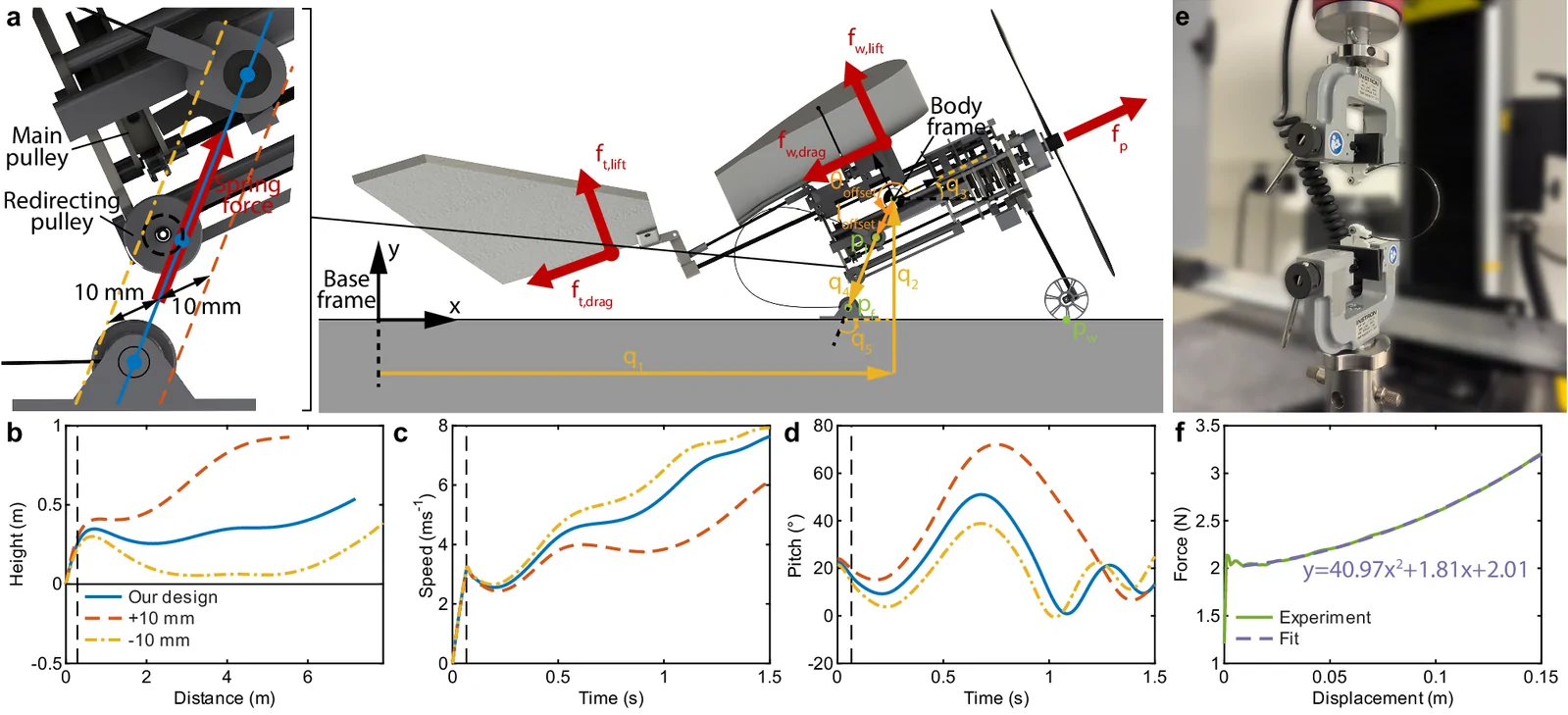
Simulation parameters and results. **a** Schematic of the model. The system is represented using five generalized coordinates (yellow). External forces are shown in red. We simulated two additional cases in which the spring force vector was shifted forward by 10 mm (orange dashed line) and backward (yellow dash-dotted line) along the body. **b** Simulated trajectory data. **c** Simulated resultant speed data. **d** Simulated pitch angle data. **e** Leg compression test setup. The carbon fiber strip used for the spring leg was installed in the Instron machine for compression testing. **f** Compression test results and corresponding fitted line. The experimental data were measured five times and averaged. The fitted line was estimated using a second-order equation.

The proper direction of the jumping force vector generated by the spring leg is critical to achieving a successful jumping take-off. This is because aligning the force vector either in front of or behind the center of mass generates a pitching moment, causing the platform to tilt upward or downward, respectively. Jumping platforms with a single actuator, as well as locusts, often encounter this challenge^40,43,44^. Excessive pitching moment may bring the drone into a stalled condition, preventing a smooth transition from jump to flight. In our single-actuator platform, the jumping force vector aligns with the line connecting the rotational joint of the leg to the redirecting pulleys located beneath the main pulley, which pulls the fishing wire (Fig. 3a). Because the leg joint and the redirecting pulleys are fixed to the body, the force vector direction remains fixed relative to the body. This characteristic allows us to simplify and consider the bending legs as an axial compression spring with fixed orientation relative to the body (Supplementary Fig. 1). We carefully designed the hardware so that the force vector passes as closely as possible through the platform’s center of mass to prevent undesirable pitching, ensuring a stable jump. The force vector passes through near the center of mass position regardless of ground angle, but our design choice is ideal on flat ground as jumping on an angled surface may cause foot slippage and an undesired initial pitch angle.

We simulated the jumping take-off to iteratively validate our system design. We conducted simulations with the jumping force vector passing through the center of mass, as well as with two additional force vector positions (Fig. 3b–d). We intentionally placed the two additional force vectors so that they do not pass through the center of mass to observe the moments created by this misalignment. To focus on the effect of the misalignment while keeping the jumping angle constant, we shifted the force vector 10 mm backward and forward along the body. The spring force was experimentally identified and used in the simulation (Fig. 3e, f). The simulation results showed that the robot successfully performed a jump take-off and sustained flight. The robot took off at 0.066 s counting from initiation of the jump, with a resultant speed of 3.15 m/s at 16.70 degrees. In 1.5 s, the robot was able to fly 7.18 m forward and 0.54 m up. During the transition from jumping to flying, the robot lost about 0.09 m in altitude. This loss was due to the time needed for the propeller to reverse its rotational direction and accelerate for thrust generation. In the case of the force vector shifted 10 mm backwards, the pitch angle was lower than in the no-shift case after the jump. As a result, the robot could not maintain level flight and descended closer to the ground level (Fig. 3b). Shifting the force vector 10 mm forward increased the pitch angle, enabling the platform to climb after the jump (Fig. 3b) but reduced the flight speed (Fig. 3c). These results suggest that jumping take-off result is sensitive to the alignment of the spring leg force vector with the center of mass, as a relatively small variation of ±10 mm results in substantial differences in the initial flight height. All three cases can be considered successful take-offs, but any further misalignment of the spring leg force may lead to a crash or flipping due to excessive pitching moments, as experimentally shown in Supplementary Fig. 2 and Supplementary Movie 1.

### Jumping take-off versus runway take-off

**Fig. 4:**
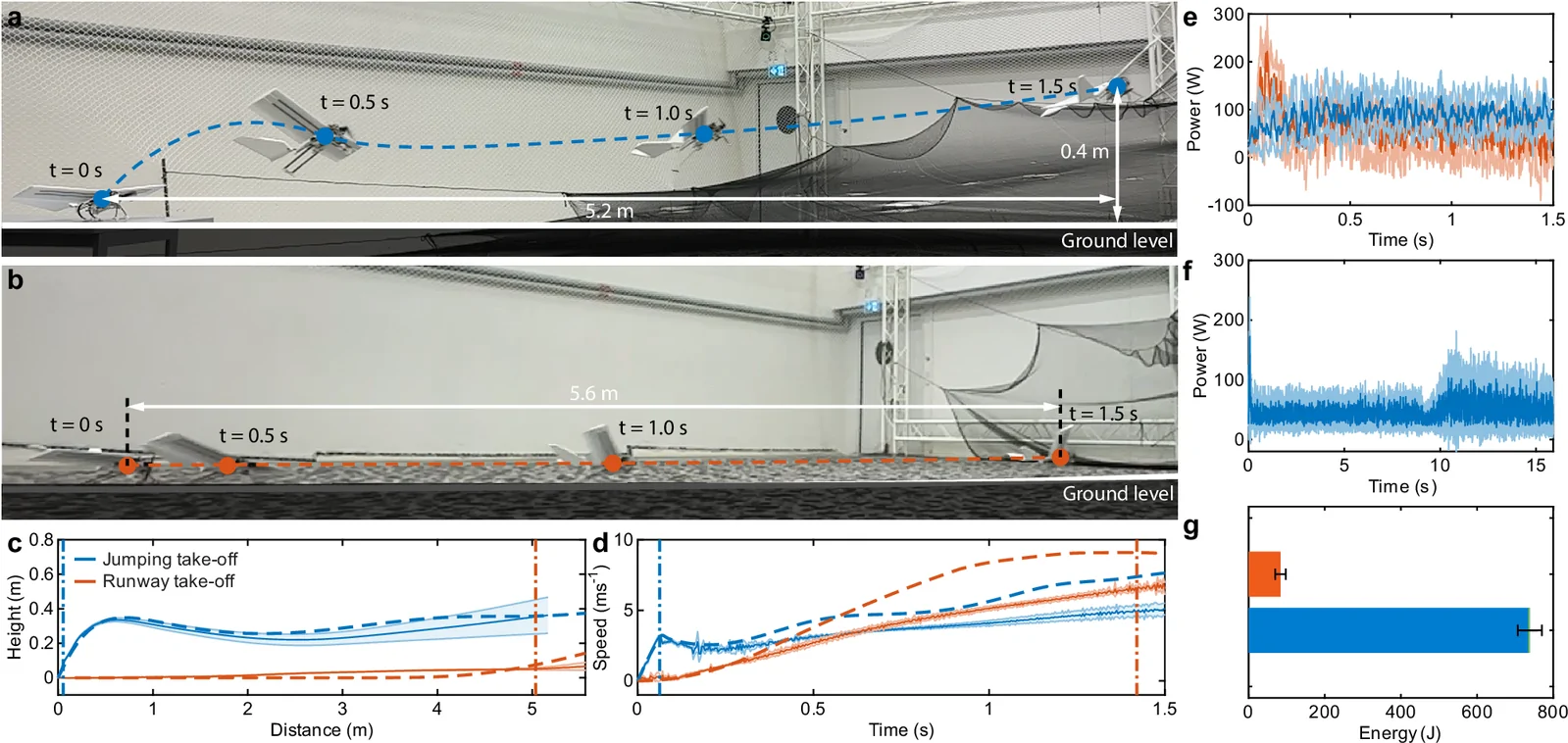
Jumping take-off and runway take-off. **a** Sequential snapshots of the jumping take-off. To ensure safety, a net was placed in front of ATROSA during both take-off experiments to reduce the impact upon landing. **b** Sequential snapshots of the runway take-off. **c** Trajectories of the jumping and runway take-offs. **d** Resultant speeds of the jumping and runway take-offs. **e** Power consumption during take-off. Blue represents jumping take-off data, while red represents runway take-off data. The solid line and shaded region indicate the mean value and standard deviation, respectively (*n* = 5). Vertical lines mark the take-off moments, and dashed lines represent simulation results. **f** Power consumption during the energy loading process of the jumping take-off. **g** Energy consumption. The thin green layer in the jumping take-off energy consumption indicates the energy consumed purely during the jump, and the rest is consumed during the loading process.

We conducted both jumping take-off (Fig. 4a and Supplementary Movie 2) and runway take-off (Fig. 4b and Supplementary Movie 3) experiments with ATROSA to compare the advantages and disadvantages of each strategy. For the runway take-off, ATROSA was programmed to generate the same level of thrust as in the jumping take-off when prompted by the RC transmitter. The experimental and simulated results are also compared in Fig. 4c, d, in which the experimental data are indicated with solid lines and simulated data are indicated with dashed lines.

ATROSA could jump for take-off and gain a height of 0.08 m at the moment of take-off and of 0.34 m eventually from the jump, highlighting its potential to take-off with obstacles ahead. Meanwhile, runway take-off required 5.04 m (11.11 body lengths) of horizontal distance to take off and gained a height of 0.05 m after 1.42 s (Fig. 4c). The jump took the robot to a speed of 3.15 m/s to initiate flight (Fig. 4d), and the runway take-off enabled ATROSA to take off at 6.43 m/s (2.04 times faster). This higher speed during runway take-off allows for a relatively more stable take-off. The higher speed in the runway take-off may result from a lower pitch angle during rolling, which enables more effective use of the propeller thrust for forward acceleration compared to the jumping take-off. The general trends of the experimental trajectory and speed data (Fig. 4c, d) closely matched the experimental results (Fig. 3b, c, and d). However, the actual platform exhibited lower speed performance, possibly due to less efficient aerodynamic characteristics of ATROSA.

Jumping take-off and runway take-off consumed 3.30 J and 83.73 J (Fig. 4e), respectively, but in the jumping take-off, storing energy in the spring leg required extra energy input. The loading consumed 734.8 J in 15.9 s (Fig. 4f). The energy outputs, the sum of kinetic energy and potential energy at take-off, of jumping take-off and runway take-off were 1.57 J and 5.93 J, respectively. By defining energy efficiency as the ratio of energy input (electrical energy consumption) to energy output (the sum of kinetic energy and potential energy) at take-off, energy efficiencies of the jumping and runway take-offs at take-off are 0.2% and 7.1%, respectively.

The experimental results present the trade-offs between jumping and runway take-off strategies. Jumping take-off offers advantages in space efficiency as this in-place strategy is less dependent on the ground conditions of the surroundings. The height gain of 0.34 m (0.76 body lengths) from the jump allows ATROSA to initiate flight even when obstacles are closely placed. However, the jumping take-off consumes 8.8 times as much energy as runway take-off, despite achieving a lower take-off speed. Therefore, runway take-off may be the preferred strategy under ideal conditions with designated infrastructure. Nevertheless, the ability to employ both take-off strategies enables flexible operation, allowing take-off in cluttered environments at the cost of increased energy consumption when necessary.

### Ground traversal capability

**Fig. 5:**
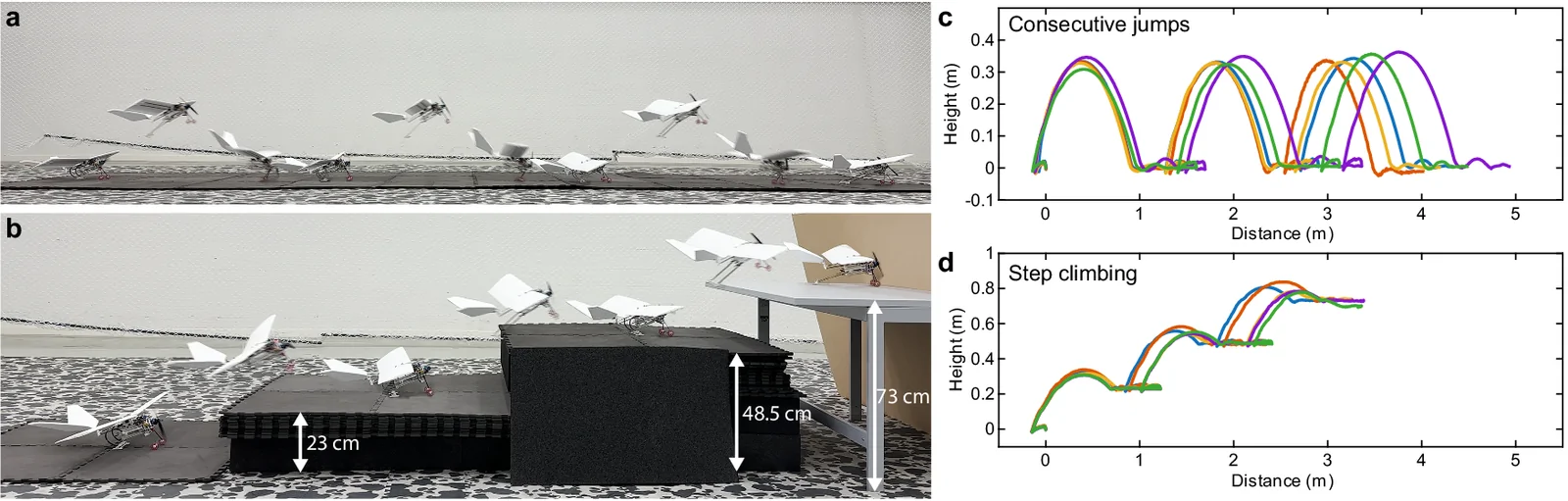
Ground locomotion by consecutive jumps. **a** Consecutive jumps on flat terrain. ATROSA is capable of traveling on the ground using consecutive jumps. **b** Step climbing by jumping. **c** Experimental trajectory data of consecutive jumps on flat ground across five trials. **d** Experimental trajectory data of step climbing across five trials.

The jump is not only a means of transitioning to aerial locomotion but can also be repeated for ground traversal. To validate the ground traversal capability of ATROSA, we conducted experiments on consecutive forward jumps on flat terrain (Fig. 5a and Supplementary Movie 4) and step climbing (Fig. 5B and Supplementary Movie 5). In these experiments, ATROSA was programmed to stop actuator rotation upon detecting a jump and to reload the spring leg after each jump. It successfully performed three consecutive jumps within the motion capture area, covering an average distance of 4.4 meters (Fig. 5c), with each jump covering approximately 1.47 m (3.7 body lengths). The average jump height was about 0.34 meters. For the step climbing experiments, we set up three steps with height differences (Fig. 5b, d). With three consecutive jumps, ATROSA successfully climbed a total height of 73 cm from its initial position.

### Outdoor operation

**Fig. 6:**
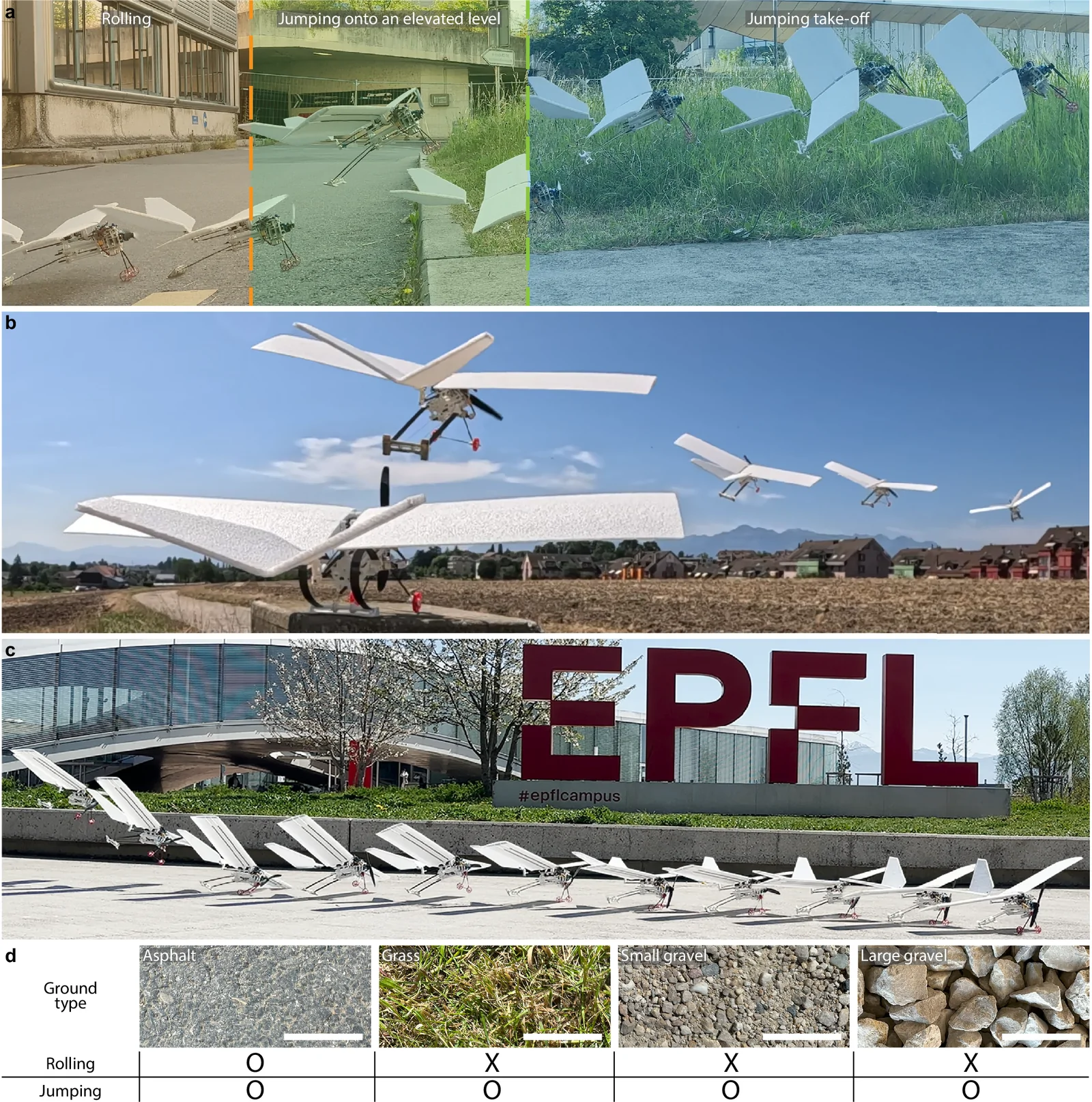
Combination of multiple locomotion modes and outdoor experiments. **a** Combination of multiple locomotion modes. ATROSA can taxi to move forward on smooth surfaces. It clears a step with a jump and, in the end, performs a jumping take-off to transition into flight mode when a runway take-off is not feasible due to limited space. **b** Outdoor jumping take-off and flight. **c** Outdoor landing. With its front wheels, ATROSA is capable of landing safely. **d** Ground operation availability based on the outdoor ground type. Scale bars, 5 cm.

ATROSA was taken out of the lab and tested outdoors in three separate tasks: a mission scenario requiring different locomotion modes, flight, and landing. In the mission scenario, ATROSA executed rolling, jumping, and taking off sequentially to traverse different types of terrains (Fig. 6a and Supplementary Movie 6). On flat terrain without obstacles ahead, ATROSA used rolling to move forward. Upon encountering a curb, which cannot be climbed by rolling, ATROSA successfully jumped onto the curb to proceed. Beyond the curb, the terrain transitioned to grass, ending in a field of tall grass that was impassable by either jumping or rolling. ATROSA performed a jumping take-off to fly over the high grass field.

The outdoor flight and landing tests are presented in Fig. 6b, c, respectively. In these tests, ATROSA was manually controlled by a human pilot. In outdoor conditions, ATROSA successfully performed jumping take-off and maintained stable flight under manual control (Supplementary Movie 7). Since autonomous flight control is not yet implemented, landing was tested separately. Landing was also successfully executed on a paved road. For video documentation, ATROSA was placed on an elevated surface, where it took off by rolling and immediately landed (Supplementary Movie 8). These experiments highlight its potential for outdoor use.

The ground locomotion performance on different ground types was evaluated by testing both rolling and jumping locomotion modes across four terrain conditions (Fig. 6d): asphalt, grass, small gravel, and large gravel. These terrains were selected because they are highly likely to be encountered during outdoor operations and have distinct characteristics in roughness, compliance, and unevenness. On asphalt, ATROSA was able to perform both rolling and jumping, as the flat and smooth surface is favorable for wheeled locomotion and provides sufficient friction for jumping (Supplementary Movie 9). Unlike paved asphalt roads, grass fields are uneven and composed of compliant grass. On this terrain, ATROSA could only perform jumping, as the wheels and foot became trapped in the gaps between the grass during rolling attempts (Supplementary Movie 9). The terrain covered with small gravel reduced the performance of both rolling and jumping (Supplementary Movie 9). During rolling, the bumpy surface formed by small gravels hindered the wheels and foot from moving forward. Also, ATROSA achieved shorter jump distances and lower heights due to energy loss caused by slippage between the small and light gravels and the ground. On terrain covered with large gravel, rolling failed for the same reason as on the small gravel terrain. However, ATROSA jumped higher and farther than it did on small gravel, as the larger gravel did not slip from the ground as easily as the smaller gravel. The uneven surface, however, caused ATROSA to jump at undesired angles, resulting in less stable landings compared to jumps on other terrains.

## Discussion

This work introduces a new robotic platform, ATROSA, capable of multimodal locomotion and transition enabled by a single actuator and a novel direction-selective transmission. ATROSA successfully showed ground traversal with jumping and rolling as well as jumping and runway take-offs. The experimental results suggest that while runway take-off is more energy-efficient, it requires 11.11 body lengths of flat, unobstructed space. In contrast, jumping take-off allows for in-place take-off with a 0.34 m height gain, offering potential for obstacle clearance during take-off. Additionally, with the same jumping strategy, ATROSA performed consecutive jumps on flat terrain and steps. ATROSA also combined rolling, step climbing, and jumping take-off to complete an outdoor mission scenario and performed flight and landing. Ground traversal on various commonly observed outdoor terrains was also presented. These experiments show the potential of ATROSA for deployment in diverse environments.

Although ATROSA has successfully performed various types of missions, there is room for improvement, particularly in energy efficiency. Jumping take-off enables ATROSA to operate in environments where a runway or launcher is unavailable. However, the energy efficiency of its jumping take-off (0.2%) is lower than that of RAVEN (4.8%)^35^ and its own runway take-off (7.1%). This low efficiency is due to the high energy consumption during spring leg loading. Our tests of two different spring-loading procedures (Supplementary Fig. 3) showed that tuning the loading procedure reduced energy consumption by 33.4%. Further optimization of the spring-loading procedure could enhance energy efficiency. Additionally, reducing friction in the mechanical system could further improve energy efficiency for the spring loading. Although many platforms share similar energy storage strategies, the energy efficiency of energy-storing systems varies across platforms^40,42,45–49^, and the differences may partially stem from friction. Careful selection of materials, refinement of manufacturing processes, and embedding bearings may reduce energy loss during the energy storage phase.

For energy-efficient locomotion on flat ground, where both rolling and jumping are available, the choice between the two modes depends on surface conditions such as roughness or slipperiness, as rolling is strongly affected by ground properties (Fig. 6). The cost of transport (CoT) enables a fair comparison between locomotion modes with different speeds and power consumption. With the faster and more energy efficient loading procedure (Supplementary Fig. 3), consecutive jumps allows ATROSA to travel at an average speed of 0.13 m/s with an average power consumption of 46.6 W. Rolling instead reaches a maximum speed of 6.43 m/s before take-off with an average power consumption of 51.1 W. Based on these values, the CoT of consecutive jumping and of rolling is 130.7 and 2.9, respectively (lower CoT indicates higher efficiency). As rolling speed decreases, CoT increases and matches that of consecutive jumping at 0.14 m/s. Therefore, if ground conditions hinder rolling and reduce its speed below 0.14 m/s, consecutive jumping becomes the more energy-efficient mode. Increasing the wheel size would improve obstacle clearance during rolling and, consequently, energy efficiency. However, the wheel size is constrained by the distance between the wheels and the propeller; therefore, the margin for improvement is limited.

Our current design limits the platform to storing and releasing fixed amounts of energy, resulting in discrete take-off energy efficiencies across two locomotion modes: jumping and rolling. By introducing a release mechanism, the system could adopt a more flexible take-off strategy that combines both modes and achieves a spectrum of energy efficiency during take-off. This spectrum could allow the robot to select an appropriate take-off strategy based on the available runway length. It could pre-store elastic energy and accelerate by rolling, then release the stored energy to jump and take off even before reaching the required take-off speed.

In the future, we envision robots like ATROSA being deployed for practical applications. For example, they could fly to hard-to-reach areas, such as mountainous regions where ground access is inefficient. After landing near the target location, the robot could switch to an appropriate ground-based locomotion mode to reach the point of interest. Once the mission is complete, it could take off again using a jumping maneuver and continue to the next destination without human intervention. Achieving this level of autonomy will require advancements in control systems. While this work showed a sequence of combined terrestrial locomotion modes, long-distance flight and flight-to-landing transitions were not shown due to the challenges of manual control. However, when scaled up, our single-actuator-based design may offer advantages in carrying heavier payloads, enabling the integration of additional sensors and greater computational resources needed for autonomous operation with advanced control schemes.

## Methods

### Manufacturing and assembling process

The majority of the custom parts were either 3D printed using polylactic acid (PLA) or thermoplastic polyurethane (TPU) with an Ultimaker 5S, or cut to the required lengths from round or square carbon fiber rods. Among the 3D-printed components, the parts connecting the main body to the wheels and the wheels themselves were printed with TPU to absorb landing impacts. All other parts were printed using PLA. The assembled parts were mostly fixed using structural methods instead of screws, to save time during assembly and reduce weight.

A propeller (GWS 8040) was mounted on the front of the BLDC motor (AT2303 KV1500 Short Shaft), while a metal shaft was attached to the opposite side to transmit power to the rest of the system. At the end of this shaft, a pinion gear with an embedded freewheel (HF0306-KF-L564) was inserted. The motor itself was secured to a 3D-printed base. On the other side of this base, five carbon fiber columns having 2 mm outer diameter were inserted to support the rest of the rotational direction-dependent transmission system. Five 3D-printed plates, each with holes for the carbon fiber columns, were axially stacked along the columns to house the gearbox, spring release mechanism, and pulley. The spacing between these plates was maintained using 3D-printed column spacers, which slid over the carbon fiber columns to resist compression. Additionally, four 3 mm × 3 mm carbon fiber square rods were used to attach various appendages to the main body. Both the plates and the appendages had matching 3 mm × 3 mm square holes. The appendages were inserted from radial direction and positioned between the plates with the square holes aligned, and the rods were inserted through these aligned holes to secure them in place. Each square rod served a specific function: the top rod held the wings, tail, and the electronics mounting plate; the two side rods secured the spring legs; and the bottom rod fixed the wheels, battery, and pulleys used to redirect the fishing wire connected to the foot. This combination of axially and radially inserted components created a self-locking structure, simplifying the assembly process while ensuring stability.

The spring leg was assembled from multiple parts. To allow smooth rotation at the proximal joint, a 3 mm inner diameter bearing was installed on each side of the leg. Carbon fiber strips (Swiss-Composite Carbon-Flachprofil 3.0 mm × 0.6 mm) were glued into 3D-printed parts that enclosed the bearings. The other ends of the strips on both sides were glued into a long 3D-printed column to synchronize the motion of the legs. The foot was attached to this column with a loose press fit, allowing it to rotate freely around the column. A layer of silicone padding (Smooth-On Ecoflex 20) was attached to the bottom of the foot. A torsional spring (Durovis 06052-R) was installed between the foot and the carbon fiber strips, enabling the foot to automatically flip and adapt between jumping and rolling modes.

### Indirect rotation observation of the motor

Both sides of the BLDC motor are occupied, one with the propeller and the other with the metal shaft connected to the transmission system. This configuration does not leave room for installing a magnetic encoder and magnet directly on the motor. To address this issue, we indirectly monitored the rotation of the motor using a set of spur gears. A spur gear was attached to the metal shaft, aligned with the center of the motor. Another gear with a magnet at the center meshed with this gear with a 1:1 gear ratio. A magnetic encoder was placed above the magnet to sense the rotation of the motor.

### Jumping mechanism characterization

The specifications of the jumping mechanism, including the spring leg dimensions and main pulley diameter, were determined iteratively through simulation. When selecting the spring leg length, we prioritized its impact on body orientation. With the fixed pivot joint and the winding angle designed to align the force vector with the center of mass, the spring leg directly influences body orientation before and during bending, as shown in Fig. 2a. Shortening leg length increases the initial pitch angle, tilting the propeller more vertically and making runway take-off impractical. In contrast, extending leg length induces negative pitch, risking propeller contact with the ground, and shifts the center of mass backward, reducing aerodynamic stability. To balance these factors, an 18 cm leg length was selected, ensuring a near-horizontal propeller orientation for runway take-off while preventing ground contact of the propeller during bending.

The thickness of the carbon fiber strip was chosen to allow bending without breaking at this length. We conducted compression tests using an Instron machine to measure the force output of the selected carbon fiber strip (Fig. 3e). The test was repeated five times, and the results were averaged to generate a force-to-compressed-distance relationship, represented as a second-order equation (Fig. 3f). The force data were fitted and used as the input of the jumping take-off simulation to estimate the number of carbon fiber strips required to generate sufficient jumping force for take-off. In the simulation, we evaluated whether the jumping force was sufficient by observing if the robot maintained clearance from the ground for a given number of carbon fiber strips. This criterion was used because, after take-off, the robot experiences drops in height and speed due to the time required for the propeller to accelerate and generate thrust. We found that 6 of the carbon fiber strips are needed theoretically for the system to achieve a successful jumping take-off and maintain flight. However, the physical implementation of the drone required two additional carbon fiber strips compared to the simulation predictions in order to compensate for frictional energy loss in the pulleys. It is worth noting that due to substantial elastic deformation during bending, stiffness degradation may occur^50^ and require periodic replacement of the carbon fibers. However, we did not observe any noticeable degradation during our relatively limited number of experiments.

The required jumping force and potentially the number of carbon fiber strips also depend on the jumping angle. The propeller requires some time to accelerate and produce sufficient lift. Lower jumping angles generate higher forward velocity, but also lower initial height that cannot be compensated in time by the accelerating propeller, thus causing ground contact. In contrast, increasing the jumping angle results in a higher initial height and provides sufficient time for propeller acceleration, but it also reduces the initial forward velocity. To demonstrate this, we simulated cases in which the spring force vector was rotated by ±10° from the designed angle while still passing through the center of mass (Supplementary Fig. 4). The results show that the case with the jumping angle increased by 10° achieved a successful take-off, whereas the case with the jumping angle decreased by 10° resulted in ground contact. As the jumping angle decreases, a greater jumping force is required, potentially requiring additional carbon fiber strips.

### Disengagement mechanism dimensions

The available winding length of the fishing wire on the pulley is determined by both the diameter of the pulley and the gear reduction ratio of the disengaging gears (Fig. 1d, e). These factors are constrained by the diameter of the planetary gearbox, as the pulley and disengaging gears need to fit within the outer holding columns (Fig. 1b). Consequently, the gear reduction ratio between the planetary gearbox output and the disengaging gears was set to 1:2 (12 teeth: 24 teeth). Within the given space, we selected the diameter of the pulley to 21 mm and removed 3 teeth from the 24-tooth disengaging gear, rotating the pulley for 87.5% of one revolution cycle before the release. As a result, the pulley completes 1.75 revolutions during each activation of the jumping mechanism, pulling the fishing wire by 115.5 mm to match the simulation result.

A similar jumping performance could theoretically be achieved with an elastic element exhibiting different stiffness at different bending angles, but this introduces trade-offs in size and mass. Since the wire length is constrained to one revolution of the pulley, the pulley diameter should be adapted accordingly. While stiffer carbon fiber strips enable a reduced body diameter through a smaller pulley, the overall mass would increase due to additional gear reduction stages and the use of more robust materials. It also becomes difficult to achieve the desired jumping angle and align the force vector with the center of gravity because the strip bending angle is limited. This reduced bending angle may also increase the risk of foot slippage due to quicker release. Conversely, using less stiff strips requires a larger bending angle and longer legs. Consequently, the pulley diameter and overall body diameter must increase. As shown in Fig. 3f, the compression–force relationship follows a second-order trend. As a result, increasing compression length is more effective than increasing stiffness for storing energy. However, longer legs may cause the leg and foot to contact the tail and shift the center of mass backward, which is detrimental to flight performance. These trade-offs highlight the importance of carefully tuning stiffness, geometry, and transmission design in accordance with the system scale and desired energy storage.

### Dynamics modeling

The simulation used Lagrange formulation with five generalized coordinates (${\boldsymbol{q}}{\boldsymbol{\in }}{{\mathbb{R}}}^{5}$) defining the horizontal (${q}_{1}$) and vertical (${q}_{2}$) positions, pitch angle (${q}_{3}$), spring leg length (${q}_{4}$), and foot angle (${q}_{5}$) in a two-dimensional space. The equations used for the jumping take-off simulation is similar to those presented in refs. ^35,49^. We assumed non-slipping conditions on the foot, which are

1 $${\dot{{\bf{p}}}}_{c}={{\bf{J}}}_{{\bf{c}}}\dot{{\bf{q}}}=0$$

2 $${\ddot{{\bf{p}}}}_{c}={{\bf{J}}}_{c}\ddot{{\bf{q}}}+{\dot{{\bf{J}}}}_{c}\dot{{\bf{q}}}=0$$

where ${{\bf{p}}}_{c}$ is the position where the foot is fixed, ${{\bf{J}}}_{{\bf{c}}}$ is the Jacobian matrix for ${{\bf{p}}}_{c}$. The number of dots indicates how many times the variable is differentiated with respect to time. The Jacobian matrix is defined as

3 $${{\bf{J}}}_{{\bf{c}}}=\frac{\partial {{\bf{p}}}_{c}}{\partial {\bf{q}}}$$

For jumping take-off, the constraint was put only on the foot, thus ${{\bf{p}}}_{c}$ was same as the foot position (${{\bf{p}}}_{f}$), which is defined as

4 $${{\bf{p}}}_{f}=\left[\begin{array}{c}{q}_{1}\\ {q}_{2}\end{array}\right]+{\bf{R}}({q}_{3}+{\theta }_{{offset}})\left[\begin{array}{c}{l}_{{offset}}\\ 0\end{array}\right]+{\bf{R}}({q}_{3}+{\theta }_{{leg}})\left[\begin{array}{c}{q}_{4}\\ 0\end{array}\right]$$

where ${\theta }_{{offset}}$ is the angle of the spring leg pivot joint with respect to the body frame, ${l}_{{offset}}$ is the distance between the body center of mass and the leg pivot joint (Fig. 3a). ${\theta }_{{leg}}$ is angle of the leg compression direction. ${\bf{R}}\in {SO}(2)$ is the rotation matrix defined as

5 $${\bf{R}}(\theta )=\left[\begin{array}{cc}\cos (\theta ) & -\sin (\theta )\\ \sin (\theta ) & \cos (\theta )\end{array}\right]$$

With these conditions, the equation of motion for the jumping take-off of the platform is written as

6 $${\bf{M}}({\bf{q}})\ddot{{\bf{q}}}+{\bf{C}}({\bf{q}},\dot{{\bf{q}}})\dot{{\bf{q}}}+{\bf{g}}({\bf{q}})+{{\bf{J}}}_{c}^{T}({\bf{q}}){{\bf{f}}}_{c}={{\bf{S}}}^{T}\tau +{{\bf{f}}}_{{ext}}$$

where ${\bf{M}}$ is the mass and inertia matrix, ${\bf{C}}$ is the centrifugal and Coriolis effect matrix, ${\bf{g}}$ is the gravitational force vector, ${{\bf{f}}}_{c}$ is the reaction force vector at the foot, τ is the force from the spring leg, ${{\bf{S}}}^{T}$ converts τ to a vector for a dimension match^51^, and ${{\bf{f}}}_{{ext}}$ is the external force vector. τ is calculated based on the fitted 2^nd^ order equation obtained from the Instron experiments (Fig. 3f). ${{\bf{f}}}_{{ext}}$ is defined as

7 $${{\bf{f}}}_{{ext}}={{\bf{f}}}_{p}+{{\bf{f}}}_{w}+{{\bf{f}}}_{t}$$

where ${{\bf{f}}}_{p}$ is the thrust from the propeller, ${{\bf{f}}}_{w}$ and ${{\bf{f}}}_{t}$ are aerodynamic forces from the wings and the tail, respectively.

### Cost of transport calculation

Cost of transport (CoT) is calculated using

8 $${\rm{CoT}}=\frac{P}{{mgv}}$$

where P is power, m is mass of the system, g is gravitational acceleration, and v is forward velocity.

### Experimental setup and data collection

The prototype measured 0.45 m × 0.83 m and weighed 280 g. We used an AT2303 Short Shaft KV 1500 motor from T-motor as the primary electric motor to drive both the propeller (GWS 8040) and the spring leg. This motor was connected to the direction-selective transmission system and to the propeller. To control the motor, we needed to measure the rotation speed, but both ends of the motor axis were connected to other components, preventing the installation of a magnetic encoder directly on the axis. To measure the motor speed indirectly, we attached a gear to the motor axis and meshed it with another gear containing an embedded magnet. An AS5048A encoder was installed in front of the magnet for the speed measurement. The main microcontroller for the system was STM32 B-G431B-ESC. This controller also controlled the motor with field oriented control (FOC) based on the speed reading from the encoder. User input was given by a radio control (RC) transmitter (FrSky Taranis X9D) to a four-channel RC receiver (FrSky D4R-II). A Seeed Studio XIAO nRF52840 microcontroller was installed to detect the jump by reading the forward direction acceleration.

For all experiments, we recorded trajectory data in a room equipped with an OptiTrack motion capture system, using 26 cameras to track the position of reflective markers on the prototype at a rate of 240 Hz. A high-speed camera was also used to capture the experiment at 240 fps. Additionally, current data was collected using a Pololu ACS724 current sensor, and this data was saved to an SD card at approximately 250 Hz.

## Supplementary information

Supplementary Information

Supplementary Movie 1

Supplementary Movie 2

Supplementary Movie 3

Supplementary Movie 4

Supplementary Movie 5

Supplementary Movie 6

Supplementary Movie 7

Supplementary Movie 8

Supplementary Movie 9

## Data Availability

All data presented in the paper are available at https://doi.org/10.6084/m9.figshare.32335212.
