# Supplementary material for: Aerial and ground locomotion of winged drones powered by a single actuator
Source: Npj Robot. 2026 Jul 29;4(1):51. doi: 10.1038/s44182-026-00108-w (PMC13574711; doi:10.1038/s44182-026-00108-w)
Supplement: Supplementary file 1 [file 44182_2026_108_MOESM1_ESM.pdf]

Supplementary Information

Aerial and ground locomotion of winged drones powered by a single actuator

Won Dong Shin[1,2*], Hoang-Vu Phan[2,3], Simon L. Jeger[2], Tristan Bonato[2], Auke J. Ijspeert[4], and Dario Floreano[2]

[1]Bio-inspired Robotics and Design Laboratory, Pohang Science and Technology University, Pohang, Republic of Korea
[2]Laboratory of Intelligent Systems, École Polytechnique Fédérale de Lausanne, Lausanne, Switzerland
[3]Avian-Insect Robotics in Motion Laboratory, University of Nevada, Reno, Reno, USA
[4]Biorobotics Laboratory, École Polytechnique Fédérale de Lausanne, Lausanne, Switzerland
[*]Corresponding author's email: wdshin@postech.ac.kr

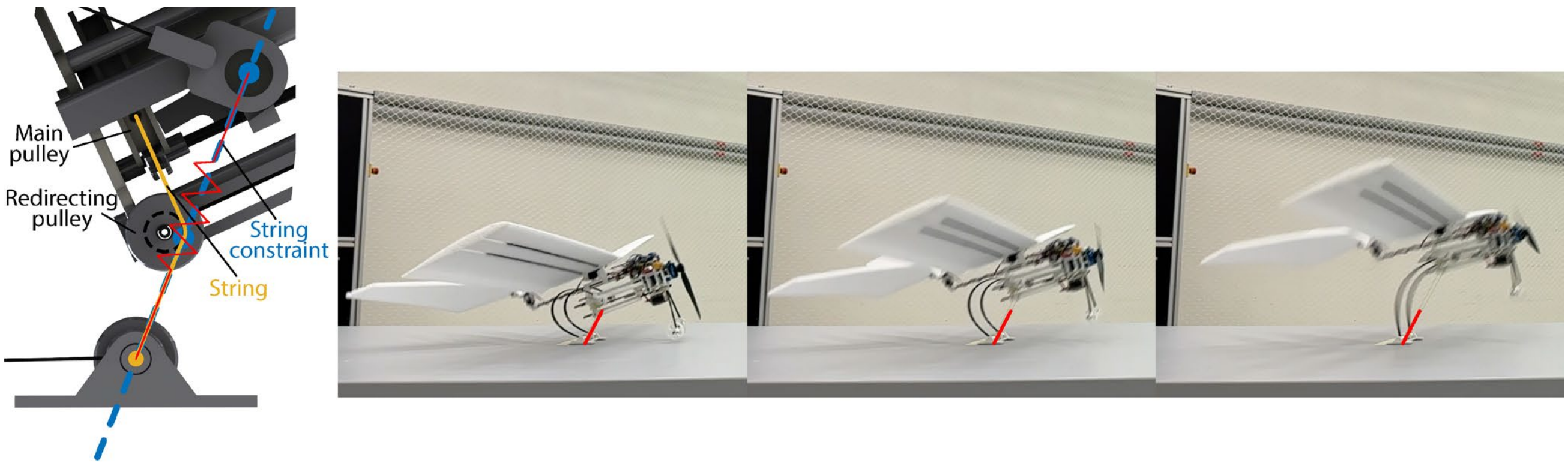

Supplementary Fig. 1 | Axial compression spring assumption of the string. The bending carbon fiber strips are treated as axially compressed springs having a second-order force equation. As illustrated in the diagram below, the positions of rotational axis of the leg and the redirecting pulley are fixed to the body. With tension in the string, the string's direction is geometrically constrained along the line (blue dashed line) connecting the two blue dots. Based on this geometric constraint, the spring force acts only in this fixed direction. The time sequential snapshots of the jump show that the spring force is acting in the fixed direction. The red lines have identical slanted angles and are well aligned with the strings.

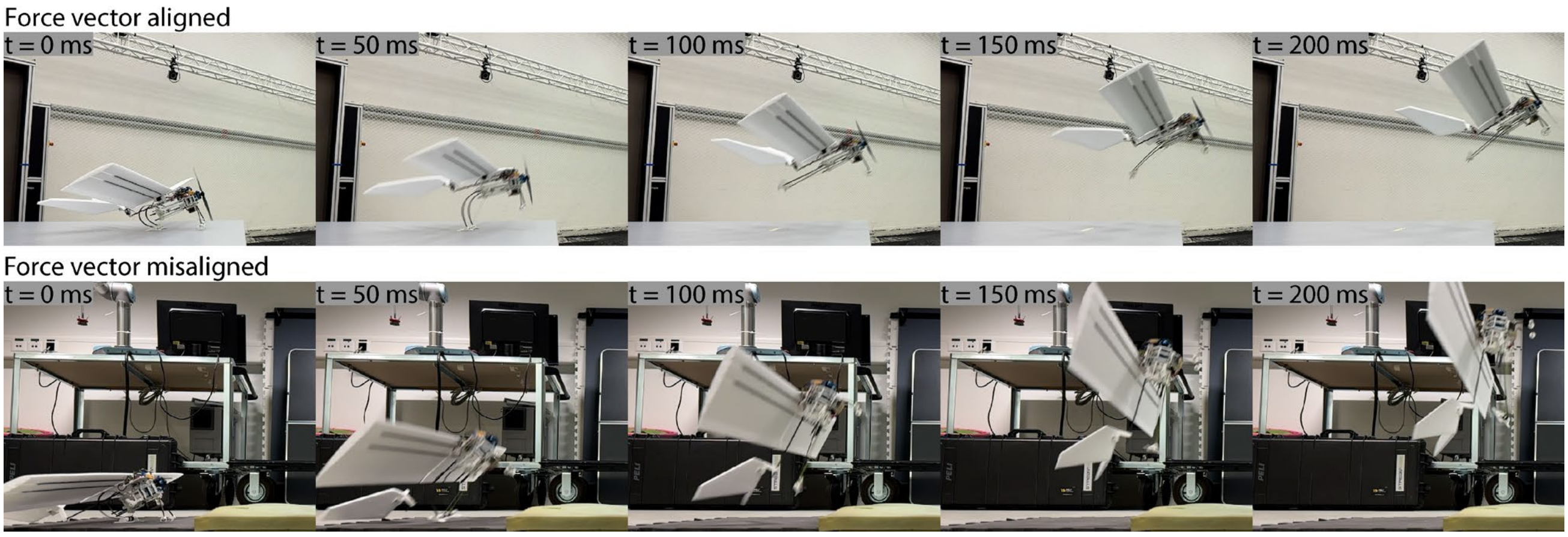

Supplementary Fig. 2 | Pitch moment created with different force vector position. When the force vector is well aligned with the center of mass of the system, the robot is able to jump without a noticeable pitching moment. However, when there is an offset between the force vector and the center of mass toward the head, an excessive pitch-up moment is generated.

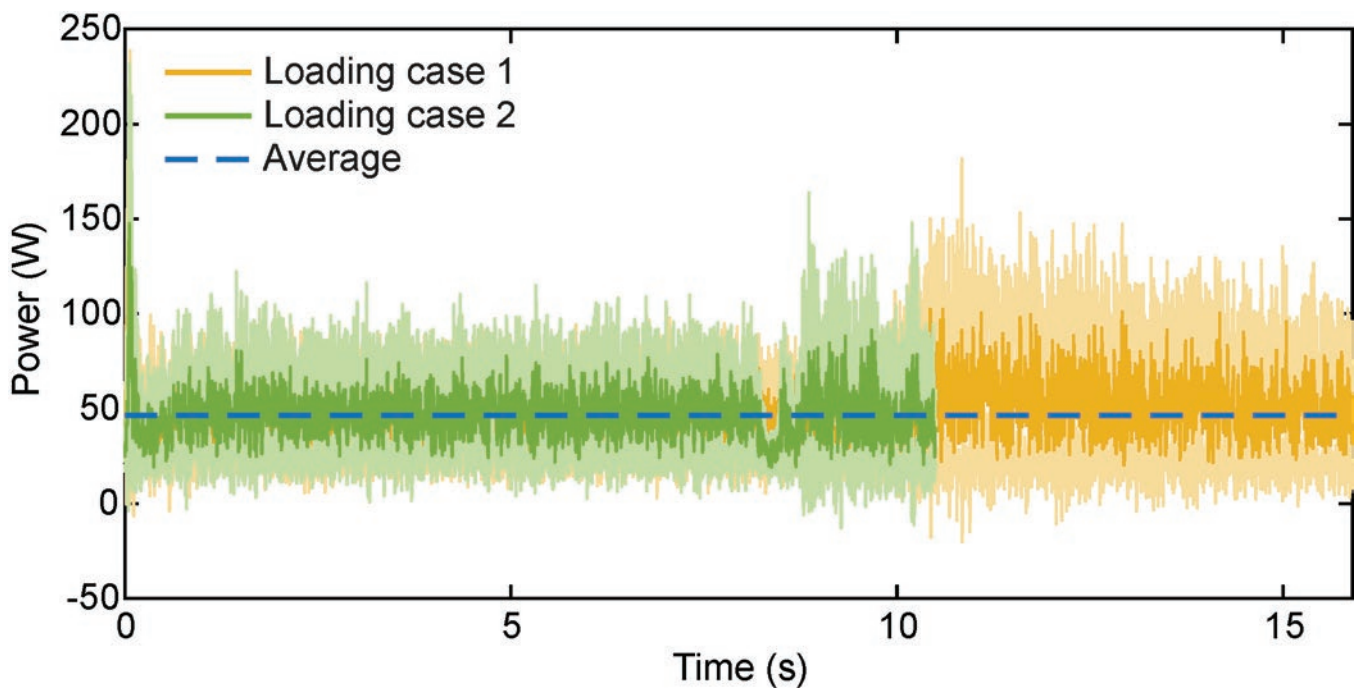

Supplementary Fig. 3 | Power consumption comparison of two different spring loading procedures. Loading case 1 corresponds to the procedure used for the jumping take-off experiment, while Loading Case 2 adjusts the motor speed to shorten the spring loading time. The average power values for loading cases 1 and 2 are 46.2 W and 46.6 W, respectively, indicating negligible variation. The dashed line denotes the mean of these two cases, 46.4 W.

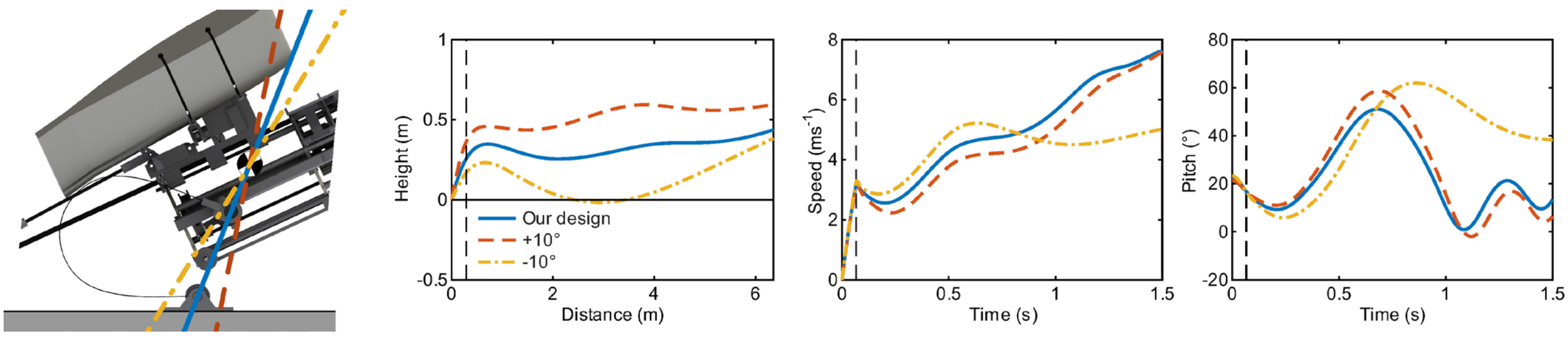

Supplementary Fig. 4 | Jumping take-off simulation with varying jumping angles. When the jumping force is applied at different angles while still passing through the center of mass, the jumping take-off performance, in terms of jumping height and speed during the early stage of take-off, varies. Jumping at a higher angle results in a successful take-off, whereas jumping at a lower angle leads to ground contact.

Description of Additional Supplementary Files

Supplementary Movie 1 –Jumping demonstration
A moment is generated when the force vector from the leg does not pass through the center of mass. A case in which the force vector is closely aligned with the center of mass and a case in which it is misaligned are compared. As expected, the misalignment creates an excessive pitching moment.

Supplementary Movie 2 –Jumping take-off
ATROSA stores elastic energy in its carbon-fiber strip legs using a pulley–actuator mechanism. When triggered, the string is automatically released through a specialized mechanical design, enabling the robot to jump. ATROSA detects the moment of jump using an IMU and immediately reverses the actuator's rotation to generate thrust, allowing a smooth transition into flight mode.

Supplementary Movie 3 – Runway take-off
ATROSA is capable of performing runway take-off using thrust from its propeller in combination with its passive wheels.

Supplementary Movie 4 – Consecutive jumps
ATROSA demonstrates consecutive jumps to traverse across the ground.

Supplementary Movie 5 – Step climbing
Using the consecutive jump strategy, ATROSA climbs a three-step staircase with a total height of 73 cm.

Supplementary Movie 6 – Outdoor mission scenario
ATROSA integrates rolling, jumping, and jumping take-off capabilities in outdoor environments to overcome various obstacles.

Supplementary Movie 7 – Outdoor flight
ATROSA demonstrates jumping take-off and sustained outdoor flight under manual control.

Supplementary Movie 8 – Outdoor landing
From a table, ATROSA flies down, lands without damage, and continues rolling using propeller thrust.

Supplementary Movie 9 – Terrestrial locomotion on different types of terrains
Jumping and rolling have been tested on four different ground conditions: asphalt, grass, small gravel, and large gravel.
